# Supplementary material for: Antibody-dependent enhancement of toxicity of myotoxin II from Bothrops asper
Source: Nat Commun. 2024 Jan 16;15:173. doi: 10.1038/s41467-023-42624-5 (PMC10791742; doi:10.1038/s41467-023-42624-5)
Supplement: Supplementary file 1 — Supplementary Information [file 41467_2023_42624_MOESM1_ESM.pdf]

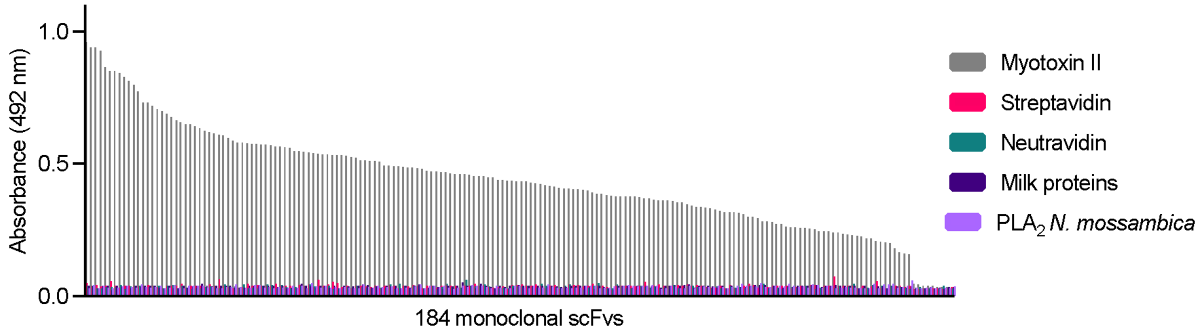

Fig. S1. Monoclonal scFv ELISA against myotoxin II, Streptavidin, Neutravidin, milk proteins, and a PLA_2 from *N. mossambica*. The majority of the scFvs bind to myotoxin II, with no scFvs binding to any of the control antigens. Binding was measured as absorbance at 492 nm. Source data are provided as a Source Data file.

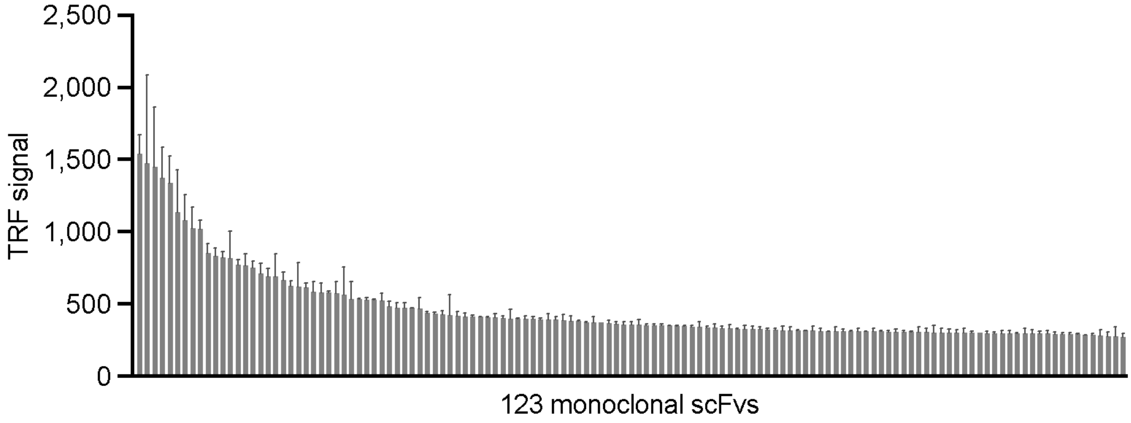

Fig. S2. Expression normalized capture (ENC) DELFIA against myotoxin II. Binding was measured using the Time-Resolved Fluorescence method on a FLUOstar Omega with excitation and emission of 337 nm and 615 nm respectively and 400 µsall integration start and integration time. Each scFv was tested in duplicates with error bars showing the standard deviation. Source data are provided as a Source Data file.

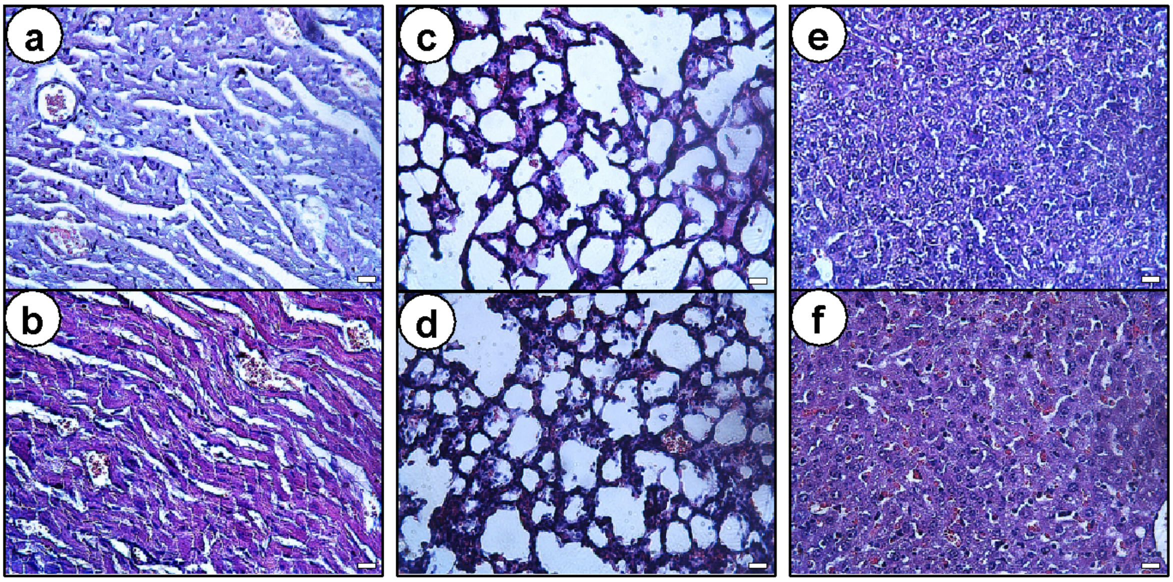

Fig. S3. Histology of different tissues from mice treated with venom only (Venom) and with venom and B12 IgG(LALA+YTE) (B12). (**a**) Heart tissue (Venom), (**b**) Heart tissue (Venom+B12), (**c**) Lung tissue (Venom), (**d**) Lung tissue (Venom+B12), (**e**) Liver tissue (Venom), (**f**) Liver tissue (Venom+B12). The scale bar in the bottom right corner of each picture is equal to 20 µm.

Table S1. Amino acid sequences of the top 6 antibodies

Antibody	Amino acid sequence
TPL0039_05_E02	QVQLLESGGGLVQPGRSLRLSCAASGFTFSSYGLHWVRQAPGKGLEWVAVISYD<br>GSNKYYADSVKGRFTISRDNSKNTLYLQMNSLRAEDTAVYYCASTLQQLDAFDIW<br>GQGTLVTVSSGGGGSGGGGSGGGASNFMLTQPHSVSESPGKTVTISCTRSSGSI<br>ASNYVQWYQQRPGTSPTIMIYEDNQRPSGVPDRFSGAIDSSSNSASLTISGLKTED<br>EADYYCQSYDSSSVVFGGGTKVTVLGQPAAASA
TPL0039_05_B12	EVQLVESGGGVVQPGRSLRLSCAASGFTFSSYGMHWVRQAPGKGLEWVAVISY<br>DGSNKYYADSVKGRFTISRDNSKNTLYLQMNSLRAEDTAVYYCAGWTEGDAFDI<br>WGQGTLVTVSSGGGGSGGGGSGGGASNFMLTQPHSVSESPGKTVTISCTRSSG<br>SIASNYVQWYQQRPGSAPSTVIYEDNRRPSGVPDRFSGSIDSASNSASLTISGLKT<br>EDEADYYCQSYDSSSVVFGGGTKITVLGQPAAASA
TPL0039_05_F04	QVQLVESGGGVVQPGRSPRLSCAASGFTFSSYGMHWVRQAPGKGLEWVAVISY<br>DGSNKYYADSVKGRFTISRDNSKNTLYLQMNSLRAEDTAVYYCAKDLEYSSSSWG<br>QGTLVTVSSGGGGSGGGGSGGGASNFMLTQPHSVSESPGKTVTISCTRSSGSIA<br>SNYVQWFQQRPGSSPTTVIYEDDQRPSGVPDRFSGSIDSSSNSASLTISGLKTED<br>EATYFCQSYDSSTVVFGGGTKVTVLGQPAAASA
TPL0039_05_G08	EVQLVESGGGVVQPGRSLRLSCAASGFTFSSYGMHWVRQAPGKGLEWVAVISY<br>DGSNKYYADSVKGRFTISRDNSKNTLYLQMNSLRAEDTAVYYCARTNYDSGDAFD<br>IWGQGTLVTVSSGGGGSGGGGSGGGASNFMLTQPHSVSESPGKTVTISCTRSS<br>GSIASNYVQWYQQRPGSSPTTVIYEDNQRPSGVPDRFSGSIDSSSNSASLTISGLK<br>TEDEADYYCQSYDSSTVVFGGGTKLTVLGQPAAASA
TPL0039_05_B04	EVQLLESGGGVVQPGRSLRLSCAASGFTFSSYGMHWVRQAPGKGLEWVAVISYD<br>GSNKYYADSVKGRFTISRDNSKNTLYLQMNSLRAEDTAVYYCARVGENEAFDIWG<br>QGTMVTVSSGGGGSGGGGSGGGASNFMLTQPHSVSESPGKTVTISCTRSSGSIA<br>RNYVQWYQQRPGSAPTTVIYEDNQRPSGVPDRFSGSIDSSSNSASLTISGLKTED<br>EADYYCQSYDSSTVVFGGGTKLTVLGQPAAASA
TPL0039_05_A03	QVQLVESGGGLAKPGRSLRLSCAVSGFTFSSYGMHWVRQAPGKGLEWVAVISYD<br>GSNKYYADSVKGRFTISRDNSKNTLYLQMNSLRAEDTAVYYCASTLQQLDAFDIW<br>GQGTMVTVSSGGGGSGGGGSGGGASNFMLTQPHSVSESPGKTITISCTRSSGSI<br>ASNYVQWYQQRPGSVPTPVIYEDDRRPSGVPDRFSGSIDSSSNSASLTISGLKTE<br>DEADYYCQSYDSSSVVFGGGTKITVLGQPAAASA
