# Supplementary material for: Antibody-dependent enhancement of toxicity of myotoxin II from Bothrops asper
Source: Nat Commun. 2024 Jan 16;15:173. doi: 10.1038/s41467-023-42624-5 (PMC10791742; doi:10.1038/s41467-023-42624-5)
Supplement: Supplementary file 2 — Reporting Summary [file 41467_2023_42624_MOESM2_ESM.pdf]

nature portfolio

Corresponding author(s): Andreas H. Laustsen and Bruno Lomonte

Last updated by author(s): Oct 13, 2023

# Reporting Summary

Nature Portfolio wishes to improve the reproducibility of the work that we publish. This form provides structure for consistency and transparency in reporting. For further information on Nature Portfolio policies, see our Editorial Policies and the Editorial Policy Checklist.

## Statistics

For all statistical analyses, confirm that the following items are present in the figure legend, table legend, main text, or Methods section.

| n/a | Confirmed | |
|---|---|---|
| ☐ | ☒ | The exact sample size (*n*) for each experimental group/condition, given as a discrete number and unit of measurement |
| ☒ | ☐ | A statement on whether measurements were taken from distinct samples or whether the same sample was measured repeatedly |
| ☐ | ☒ | The statistical test(s) used AND whether they are one- or two-sided<br>*Only common tests should be described solely by name; describe more complex techniques in the Methods section.* |
| ☒ | ☐ | A description of all covariates tested |
| ☐ | ☒ | A description of any assumptions or corrections, such as tests of normality and adjustment for multiple comparisons |
| ☐ | ☒ | A full description of the statistical parameters including central tendency (e.g. means) or other basic estimates (e.g. regression coefficient) AND variation (e.g. standard deviation) or associated estimates of uncertainty (e.g. confidence intervals) |
| ☐ | ☒ | For null hypothesis testing, the test statistic (e.g. $F$, $t$, $r$) with confidence intervals, effect sizes, degrees of freedom and $P$ value noted<br>*Give P values as exact values whenever suitable.* |
| ☒ | ☐ | For Bayesian analysis, information on the choice of priors and Markov chain Monte Carlo settings |
| ☒ | ☐ | For hierarchical and complex designs, identification of the appropriate level for tests and full reporting of outcomes |
| ☒ | ☐ | Estimates of effect sizes (e.g. Cohen's $d$, Pearson's $r$), indicating how they were calculated |

*Our web collection on statistics for biologists contains articles on many of the points above.*

## Software and code

Policy information about availability of computer code

| | |
|---|---|
| Data collection | N/A |
| Data analysis | N/A |

For manuscripts utilizing custom algorithms or software that are central to the research but not yet described in published literature, software must be made available to editors and reviewers. We strongly encourage code deposition in a community repository (e.g. GitHub). See the Nature Portfolio guidelines for submitting code & software for further information.

## Data

Policy information about availability of data

All manuscripts must include a data availability statement. This statement should provide the following information, where applicable:

- Accession codes, unique identifiers, or web links for publicly available datasets
- A description of any restrictions on data availability
- For clinical datasets or third party data, please ensure that the statement adheres to our policy

Protein sequences for the antibodies (in scFv format), TPL0039_05_E02, TPL0039_05_B12, TPL0039_05_F04, TPL0039_05_G08, TPL0039_05_B04, and TPL0039_05_A03, are available in Supplementary Table 1. The data generated in this study are provided in the Source Data file.

## Research involving human participants, their data, or biological material

Policy information about studies with human participants or human data. See also policy information about sex, gender (identity/presentation), and sexual orientation and race, ethnicity and racism.

| | |
|---|---|
| Reporting on sex and gender | N/A |
| Reporting on race, ethnicity, or other socially relevant groupings | N/A |
| Population characteristics | N/A |
| Recruitment | N/A |
| Ethics oversight | N/A |

Note that full information on the approval of the study protocol must also be provided in the manuscript.

# Field-specific reporting

Please select the one below that is the best fit for your research. If you are not sure, read the appropriate sections before making your selection.

☒ Life sciences ☐ Behavioural & social sciences ☐ Ecological, evolutionary & environmental sciences

For a reference copy of the document with all sections, see nature.com/documents/nr-reporting-summary-flat.pdf

# Life sciences study design

All studies must disclose on these points even when the disclosure is negative.

| | |
|---|---|
| Sample size | Sample sizes for in vivo experiments were chosen based on standard sample sizes involved in venom neutralisation studies with cohorts of 5 animals. |
| Data exclusions | In the data analysis of muscle damage measurements in mice, one outlier data point was identified and removed. This data point exhibited a value over three times higher than the mean of the remaining four replicates. |
| Replication | The experiment showing the first case of ADET was reproduced once (two times total). All other experiments were not reproduced but was also carried out in triplicates or quintuplicates. |
| Randomization | Not relevant in studies involving venom neutralization, as the outcome (Creatine Kinase levels due to myotoxicity) is clearly evident from measurements. |
| Blinding | Blinding is not relevant in studies involving venom neutralization, as the outcome (Creatine kinase levels due to myotoxicity) is clearly evident from measurements. |

# Reporting for specific materials, systems and methods

We require information from authors about some types of materials, experimental systems and methods used in many studies. Here, indicate whether each material, system or method listed is relevant to your study. If you are not sure if a list item applies to your research, read the appropriate section before selecting a response.

### Materials & experimental systems

| n/a | Involved in the study | |
|---|---|---|
| ☐ | ☒ | Antibodies |
| ☐ | ☒ | Eukaryotic cell lines |
| ☒ | ☐ | Palaeontology and archaeology |
| ☐ | ☒ | Animals and other organisms |
| ☒ | ☐ | Clinical data |
| ☒ | ☐ | Dual use research of concern |
| ☒ | ☐ | Plants |

### Methods

| n/a | Involved in the study | |
|---|---|---|
| ☒ | ☐ | ChIP-seq |
| ☒ | ☐ | Flow cytometry |
| ☒ | ☐ | MRI-based neuroimaging |

## Antibodies

| | |
|---|---|
| Antibodies used | Antibodies discovered by us has been disclosed as described in the data availability statement.<br>anti-FLAG M2-Peroxidase (Sigma Aldrich, #A8592) |

| | |
|---|---|
| | anti-FLAG M2 (Sigma, F1804)<br>Anti-Human IgG(Fc-specific)-Peroxidase antibody (Sigma-Aldrich, #A0170) |
| Validation | From manufacturers pages:<br><br>anti-FLAG M2-Peroxidase (Sigma Aldrich, #A8592)<br>General description<br>The Monoclonal ANTI-FLAG M2-Peroxidase is a mouse IgG antibody covalently conjugated to horseradish peroxidase ( HRP). The antibody binds to FLAG fusion proteins and recognizes the FLAG epitope at N-terminal, Met-N-terminal, C-terminal, and internal FLAG peptides.<br><br>anti-FLAG M2 antibody (Sigma, F1804)<br>Specificity:Detects a single band of protein on a Western Blot from mammalian crude cell lysates by chemiluminescent probing<br>Sensitivity Test Conforms Detects 2 ng of FLAG-BAP fusion protein by Dot Blot using Chemiluminescent Detection.<br><br>AAnti-Human IgG(Fc-specific)-Peroxidase antibody (Sigma-Aldrich, #A0170)<br>Specificity of the Anti-Human IgG (Fc specific)- Peroxidase is determined by ELISA. The conjugate is specific for human IgG (Fc fragment) when tested against human IgA, IgG (Fab and Fc fragments), IgM, Bence Jones kappa, and lambda myeloma proteins. Cross-reactivity of the antibody-conjugate is determined by ELISA. The conjugate shows no reactivity with mouse or rat IgG and and yields reduced background with mouse or rat samples. |

## Eukaryotic cell lines

Policy information about cell lines and Sex and Gender in Research

| | |
|---|---|
| Cell line source(s) | Fibroblast (neonatal; 106-0SN, Sigma Aldrich) |
| Authentication | The cell line was not authenticated |
| Mycoplasma contamination | The cell line was not tested for mycoplasma contamination. |
| Commonly misidentified lines<br>(See ICLAC register) | None |

## Animals and other research organisms

Policy information about studies involving animals; ARRIVE guidelines recommended for reporting animal research, and Sex and Gender in Research

| | |
|---|---|
| Laboratory animals | CD-1 mice, 3-4 weeks old, 18-20 g |
| Wild animals | The study did not involve wild animals |
| Reporting on sex | Both sexes |
| Field-collected samples | The study did not involve samples collected from the field |
| Ethics oversight | Mouse experiments followed ethical guidelines of the Institutional Committee for the Use and Care of Animals (CICUA, #084-17) of the University of Costa Rica |

Note that full information on the approval of the study protocol must also be provided in the manuscript.
